# Ten Year Trends in Community HIV Viral Load in Barbados: Implications for Treatment as Prevention

**DOI:** 10.1371/journal.pone.0058590

**Published:** 2013-03-08

**Authors:** R. Clive Landis, Songee Lynn Branch-Beckles, Shawna Crichlow, Ian R. Hambleton, Anton Best

**Affiliations:** 1 Chronic Disease Research Centre, The University of the West Indies, Bridgetown, Barbados; 2 Ladymeade Reference Unit, HIV/STI Programme, Ministry of Health, Bridgetown, Barbados; Fundacion Huesped, Argentina

## Abstract

**Background:**

Treatment as prevention is a paradigm in HIV medicine which describes the public health benefit of antiretroviral therapy (ART). It is based on research showing substantial reductions in the risk of HIV transmission in persons with optimally suppressed HIV-1 Viral Loads (VL). The present study describes ten year VL trends at the national HIV treatment unit and estimates VL suppression at a population level in Barbados, a Caribbean island with a population of 277,000, an estimated adult HIV prevalence of 1.2%, and served by a single treatment unit.

**Methods:**

The national HIV treatment centre of the Barbados Ministry of Health has a client VL database extending back to inception of the clinic in 2002 (n = 1,462 clients, n = 17,067 VL measurements). Optimal VL suppression was defined at a threshold value of ≤200 viral copies/mL.

**Results:**

Analysis of VL trends showed a statistically significant improvement in VL suppression between 2002 to 2011, from 33.6% of clients achieving the 200 copies/mL threshold in 2002 to 70.3% in 2011 (P<0.001). Taking into account the proportion of clients alive and in care and on ART, the known diagnosed HIV population in Barbados, and estimates of unknown HIV infections, this translates into an estimated 26.2% VL suppression at a population level at the end of 2010.

**Conclusions:**

We have demonstrated a significant trend towards optimal VL suppression in clients utilizing the services of the national HIV treatment program in Barbados over a 10-year period. Estimates of VL suppression at a population level are similar to reports in developed countries that applied similar methodologies and this could suggest a public health benefit of ART in minimizing the risk of sexual transmission of HIV. Continued efforts are warranted to extend HIV testing to hidden populations in Barbados and linking infected persons to care earlier in their disease.

## Introduction

The Caribbean has the second highest prevalence of HIV in the world (0.9%) outside that of sub-Saharan Africa, with 200,000 persons estimated to be living with HIV [1]. HIV/AIDS remains the leading cause of death in the 25–44 age group in the Caribbean [2]. In Barbados the overall adult prevalence of HIV is estimated at 1.2% [3]. The transmission of HIV in the Caribbean centers largely around sex, primarily driven by unprotected heterosexual intercourse [2]; [4]–[6]. Despite high levels of HIV awareness and education in the Caribbean, levels of reported condom use remain inconsistent [6]–[8]. The ability to exercise condom use by females is impacted by entrenched gender stereotypes, cultural attitudes towards sex, asymmetric power distribution within relationships, sexual violence, and unequal access to resources [4]; [5]; [9]–[12]. Given such challenges, there is growing interest in biomedical prevention measures that can complement behavioral interventions to yield greater impact in HIV prevention.

Vertical transmission from mother to child and heterosexual transmission of HIV has been shown to be suppressed by ART in direct relation to suppression of viral load [13]; [14]. The concept of treatment as prevention has gained traction with observational studies, cohort studies, the “Swiss Statement”, meta-analyses and a recent RCT demonstrating the near elimination of HIV transmission in persons receiving ART with a suppressed VL [15]–[21]. A threshold value for optimal VL suppression was proposed by the U.S Centers for Disease Control and Prevention (CDC) at ≤200 copies/mL [22]. Using this threshold value, the CDC estimated 28% of the HIV infected population in the USA had optimally suppressed VL [23]. A number of studies have calculated the predicted public health benefits accruing from suppressed community viral load [24]–[26], while the public health benefits of ART were cited in the shift of public health thinking in North America towards adopting “test and treat” (i.e. to treat HIV patients as soon as a seropositive result is confirmed, regardless of CD4 level) [27].

The Ladymeade Reference Unit (LRU) is the national HIV treatment centre of Barbados established in 2002 as part of the Government’s expanded response to the HIV epidemic. According to the 2010 Barbados HIV Surveillance Report there were 1055 patients alive and registered under the care of the LRU out of a total number of 1918 diagnosed HIV-infected persons [3]. Highly active antiretroviral therapy was instituted at the LRU in 2002 using a treatment threshold of CD4≤200 cells/mL, later raised to CD4≤350 CD4 cells/mL in 2010. The LRU client database contains VL data extending back to 2002.

The present study describes the VL trends from the LRU database 2002 to 2011 and derives an estimate of population level VL suppression at the end of 2010.

## Methods

### HIV Client Database

The Ladymeade Reference Unit (LRU) is the national HIV treatment centre of Barbados, consisting of a clinic with in-house pharmacy and an internationally accredited laboratory. Viral load data collected from clients attending the LRU between 2002–2011 as part of their routine clinical care was accessed from the Sexual Health Information Programme (SHIP) database. The secondary analysis of aggregated data performed in this study from a coded dataset containing no personal identifiers was waived by the chairman of the joint Ministry of Health/University of the West Indies Ethical Review Board (ERB) from further consideration of the ERB. The Ministry of Health does not require explicit consent from patients accessing healthcare facilities for permission to use aggregate laboratory indicators collected as part of routine patient care for the purposes of disease surveillance or programmatic monitoring and evaluation. This stance is supported by the local ERB. Data were abstracted from 1462 patients attending the LRU clinic between 01 January 2002 and 31 December 2011. VL determinations were made at 3–12 month intervals depending on individual virological control and the discretion of the treating physician. The total number of viral load measurements covering the 10 year period in the SHIP database was 17,607.

### ART Treatment Guidelines

ART treatment guidelines in Barbados indicate that ART should be started as early as possible for symptomatic patients and for asymptomatic patients when the CD4 count closely approaches or drops below 350 cells/mL. The treatment threshold was raised in 2010 from ≤200 cells/mL to ≤350 cells/mL.

### Viral Load Measurement

Two 4ml blood samples were collected in EDTA collection tubes (BD Biosciences, Franklin Lakes, New Jersey, USA) for each patient. Samples were centrifuged (Beckman Coulter Allegra 6R, Brea, California, USA) at 800–1600×g for 20 minutes at room temperature (18–25°C). Centrifuged plasma was transferred into sterile cryotubes (Nunc, Roskilde, Denmark) and stored at −80°C until testing. Viral load determinations were performed using the Roche Cobas Amplicor™ (Roche Diagnostics, Mannheim, Germany) from 2002 to October 2007 and Roche AmpliPrep® and TaqMan® 48 Analysers (Roche Diagnostics) from November 2007 to December 2011, following the manufacturer’s instructions. The minimum detection limit was 40 copies/mLfor the Roche Cobas Amplicor™ and 20 copies/mLfor the Roche TaqMan® 48.

### Estimates of HIV Prevalence

These were calculated using ante-natal clinic and diagnosed HIV infection data to estimate national prevalence with the EPP software model [3]; [28]. Incidence trends from the EPP model were used as one of the inputs for the Spectrum software model to provide an estimate of HIV infection trends in the country.

### Statistical Analysis of Viral Load Trends

VL threshholds for optimal suppression (≤200 copies/ml) and high viremia (≥100,000 copies/ml) were adopted from the CDC guidance document on community viral load calculation [22]. For each year we reported median VL and interquartile range (IQR), the proportion of clients with optimal VL suppression (≤200 copies/ml), and the proportion with high viremia (≥100,000 copies/ml), and recording whether the patient was receiving ART therapy at the time of each VL measurement (yes or no). To determine longitudinal VL trends, we used a random effects logistic regression to provide a test for trend (between 2002 and 2011) for the proportion of clients considered controlled (≤200 copies/ml) when receiving at least two ART treatments over a 12-month period, and excluding viral load measurements prior to ART. Graphic representation of yearly VL was performed using kernel density plots, with these plots using VL measurements from all clients in the database receiving ART. Due to the skewed distribution of the VL data, all analyses use VL on a common log scale. Statistical significance was assumed at P<0.05 although exact P-values were presented at all times to clarify the strength of the statistical relationships. Statistical analysis was performed using Stata 12 statistical software (Stata Corp., College Station, TX).

## Results

### Client Population

There were 1,462 clients attending the LRU receiving a viral load measurement at least once between 01 January 2002 and 31 December 2011 (**Table 1**). 803 or 54.9% were men. There were 17,607 individual viral load measurements, for an average of 12.04 measurements per individual (SD 8.02 measurements). Patients registered for longer had more visits – ranging from an average of 19.79 visits among those registered in 2002 to 1.9 visits among those registered in 2011. There was an average of 2.4 visits per year per patient (SD 1.06 visits).

**Table 1:** Number of patients measured for viral load by sex and year between 2002 and 2011.

	WOMEN			MEN		
YEAR	Number of patients enrolled	Average visits since enrolment (mean, SD)	Average visits in year (mean, SD)	Number of patients enrolled	Average visits since enrolment (mean, SD)	Average visits in year (mean, SD)
2002	75	19.16 (8.41)	1.63 (0.73)	81	20.38 (8.19)	1.65 (0.74)
2003	56	17.41 (6.99)	2.25 (1.16)	89	17.13 (8.69)	2.26 (1.41)
2004	166	16.05 (7.17)	2.71 (1.12)	174	15.71 (7.32)	2.8 (1.13)
2005	76	11.26 (6.52)	2.62 (1.14)	48	12.83 (6.56)	2.66 (1.1)
2006	73	9.18 (5.4)	2.12 (0.96)	87	10.53 (6.07)	2.27 (0.96)
2007	54	9.37 (4.96)	2.13 (0.93)	74	9.86 (4.71)	2.18 (0.99)
2008	54	8.31 (4.43)	2.46 (1.11)	78	9.21 (3.6)	2.66 (1.25)
2009	39	7.21 (3.23)	2.47 (1.02)	46	6.39 (3.26)	2.65 (1.11)
2010	29	4 (2.05)	2.4 (0.92)	65	4.35 (1.75)	2.41 (0.95)
2011	37	2 (1.03)	2.26 (0.91)	61	1.84 (0.9)	2.23 (0.85)
**Period**	**659**	**12.18 (7.89)**		**803**	**11.93 (8.12)**	

### Viral Load Trends

**Figure 1:**
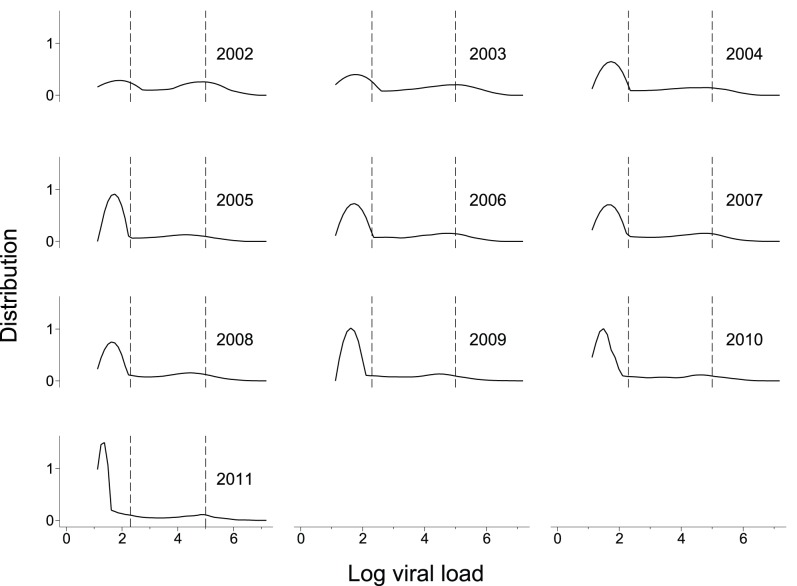
Distribution of viral load measurements over time among patients receiving ART at any time. VL distribution is represented as a kernel density plot on a log scale to highlight the bimodal distribution of the data set. Vertical dotted lines on each plot indicate the CDC thresholds for optimal suppression and high viremia (≤200 and ≥100,000 copies/mL respectively).

**Figure 2:**
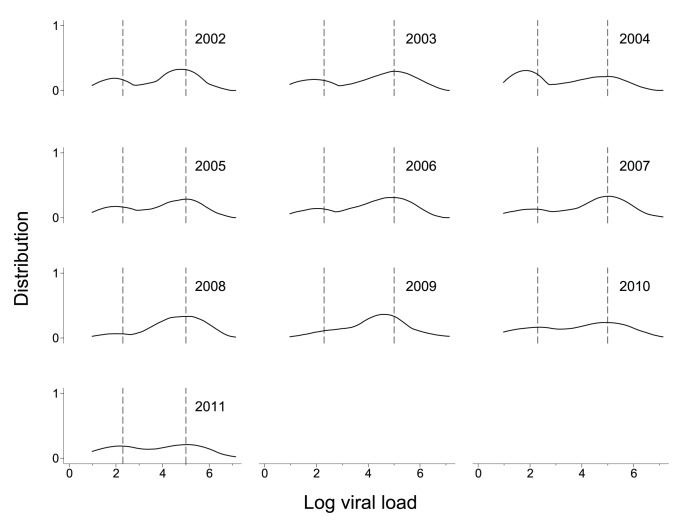
Distribution of first viral load measurement among patients receiving ART at any time. VL distribution is represented as a kernel density plot on a log scale to highlight the bimodal distribution of the data set. Vertical dotted lines on each plot indicate the CDC thresholds for optimal suppression and high viremia (≤200 and ≥100,000 copies/mL respectively).

Median log VL decreased from 3.8 in 2002 to 1.3 in 2011. This equates to a median of 6,710 copies/mL in 2002 and 20 copies/mL in 2011. Among patients on ART the median change was from 1.9 to 1.3 (78 copies/mL down to 20 copies/mL), and among those not on ART the change was from 4.8 to 2.5 (56,650 copies/mL down to 282 copies/mL). **Figure 1** shows the 10 yr VL trends for all clients in the database receiving ART (n = 15,266 data points). This figure illustrates the bimodal nature of VL distribution in the patient population and the steady improvement of VL suppression over time. **Table 2** shows the median VL and interquartile range for each year. The odds of having a controlled viral load (<200 copies/ml) increased between 2002 and 2011, with the chance of VL control improving by 13% in each consecutive year (Odds ratio 1.13, 95% CI 1.10–1.17, P<0.001, data not shown). The improvement in VL suppression was not mirrored by improvements in first ever VL measurement, which served as a proxy for disease progression at the point of enrolment into the HIV treatment program (**Figure 2**).

**Table 2:** Viral load summary between 2002 and 2011.

	ALL PATIENTS		PATIENTS ON ART		PATIENTS NOT ON ART	
Year	N	Median log viral load (copies/mL) (IQR)	N	Median log viral load (copies/mL) (IQR)	N	Median log viral load (copies/mL) (IQR)
2002	256	3.8 (1.7–4.9)	132	1.9 (1.7–3.7)	124	4.8 (3.9–5)
2003	623	3.2 (1.7–4.7)	394	1.7 (1.7–3.6)	229	4.5 (3.9–5.1)
2004	1,669	2 (1.7–4.1)	1,164	1.7 (1.7–2.6)	505	4.2 (3.4–5)
2005	1,774	1.7 (1.7–3.5)	1,332	1.7 (1.7–1.7)	442	3.9 (3.1–4.5)
2006	1,721	1.7 (1.7–4)	1,335	1.7 (1.7–2.6)	386	4.1 (3.3–4.9)
2007	1,861	1.7 (1.6–4)	1,419	1.7 (1.6–2.6)	442	4.1 (3.2–4.8)
2008	2,394	1.7 (1.6–3.9)	1,792	1.6 (1.6–2.4)	602	4.1 (3–4.7)
2009	2,536	1.6 (1.6–3.5)	1,929	1.6 (1.6–2.2)	607	3.8 (2.4–4.5)
2010	2,407	1.6 (1.3–3.3)	1,882	1.6 (1.3–2.2)	525	3.4 (1.6–4.5)
2011	2,366	1.3 (1.3–2.9)	1,837	1.3 (1.3–2.2)	529	2.5 (1.3–4.3)

### Virological Suppression

The proportion of patients falling on either side of the “high viremia” and “optimal suppression” thresholds is shown in **Table 3**. Whereas virological control was relatively poor in 2002, with just 33.6% of the client population achieving the 200 copies/mL threshold, this improved to 70.3% of clients in 2011. The percentage controlled among those on ART increased from 58.3% to 76.7%, and the percentage controlled of those not on ART increased from 7.3% to 48.0%. A corresponding drop in the proportion of VL measurements above the “high viremia” threshold was noted, falling from 16.4% in 2002 to 5.7% in 2011 for the whole client population.

**Table 3:** Proportion of clients having a suppressed viral load (<200 copies/ml) between 2002 to 2011.

	ALL PATIENTS		PATIENTS ON ART		PATIENTS NOT ON ART	
Year	% Suppressed	% High viremia	% Suppressed	% High Viremia	% Suppressed	% High Viremia
2002	33.6	16.4	58.3	7.6	7.3	25.8
2003	43.2	18.3	64.0	11.4	7.4	30.1
2004	53.2	11.1	72.0	5.4	9.7	24.4
2005	63.1	5.4	80.0	3.7	12.4	10.6
2006	57.4	8.1	72.8	5.0	3.9	18.9
2007	57.3	8.1	72.6	5.5	8.4	16.5
2008	58.7	7.0	73.5	4.6	14.6	14.3
2009	63.3	5.4	76.1	4.7	22.7	7.7
2010	66.7	6.3	76.0	5.7	33.5	8.6
2011	70.3	5.7	76.7	5.6	48.0	6.1

### Estimating Population Level VL Suppression

VL suppression at a population level may be extrapolated based on 2010 national surveillance data and estimates of the total HIV population, using previously described methodology [23]. The total number of persons estimated to be living with HIV in Barbados in 2010 was 2323 [28]. Case-based surveillance determined that 1918 people were alive and diagnosed with HIV at 31-Dec-2010 [3]. There were 1055 people known to be alive and in-care at the LRU clinic (and with a measured viral load) at 31-Dec-2010. Of these 792 people were retained in care, where “retained in care” means 2 or more visits over the 12 month period before 31-Dec-2010 [29]. There were 648 people retained in care and on ART, and, finally, optimal virological suppression below the 200 copies/mL threshold was demonstrated in 608 persons at the end of 2010. Hence, by previous methodology to define population level VL suppression [23], we calculate VL suppression in 2010 in the Barbados HIV-infected population (known and unknown) is 26.2% ((608/2323) x 100 = 26.2%).

## Discussion

We have demonstrated a statistically significant downward trend in community VL amongst the LRU client population over a 10-year period and have estimated population wide VL suppression in Barbados for the year 2010. As noted by Gardner et al., treatment as prevention can only work effectively at population-level if a high proportion of all HIV-infected people are: (i) tested for HIV, (ii) linked to clinical care in a timely manner, (iii) retained in care, (iv) receive effective ART, and (v) adhere to treatment and are regularly monitored [26]. Barbados satisfies these criteria for patient engagement in certain respects: classified by the World Bank as a high-income country [30], Barbados has an estimated level of undiagnosed infections (21%) within a range reported for other resource-rich settings [31]–[34]. The proportion of HIV diagnosed persons linked to care (55%) remains an area of concern and is consistent with the observation that 31.7% of patients enter the program with AIDS, with 50% qualifying for immediate ART [3]. There was little evidence from first ever VL measurements for an improving trend in healthcare seeking behavior over the 10 year period. The proportion of patients retained in care (75%), on ART (82%), and achieving VL suppression (94%) is at the upper end of rates reported for developed countries [20]; [32]; [35]; [36] but consistent with the elimination of HIV positive births from Barbados since 2007 due to ART treatment of HIV positive pregnant mothers [3].

The authors are aware of only one study reporting VL suppression at a national level; a CDC report demonstrating 28% VL suppression in the US HIV population [23]. Our study was not an ecological analysis and we recognize the limitations and uncertainty in extrapolating population level VL suppression from a public clinic database, but the projected estimate of 26% national VL suppression is useful for comparing the barriers that exist between Barbados and the USA towards engagement of patients in care. In the USA notable challenges were reported in the retention of patients in care and in achieving VL suppression on ART, whereas in Barbados the biggest challenge was in linking HIV diagnosed individuals to care. Our data captures a proportion of private patients who are having VL determinations performed at the LRU laboratory. However, an estimated 2.6% of the diagnosed HIV population - patients who may be seeking private care and private labs elsewhere - is not contained in our database, offering a partial explanation to the problem of linking diagnosed cases to care. Our findings argue for a continued emphasis on destigmatizing HIV, to engage patients in care at an earlier stage of their disease, and to maintain and promote universal access with state funding for HIV treatment including the provision of ART [37]–[39]. Notwithstanding such challenges, the experience of Barbados should give hope to other Caribbean territories that VL suppression due to ART is an achievable goal. VL suppression at a population level may have played a role, in conjunction with other behavioral or structural interventions, in the decline of new cases from a peak in 2000 concurrent with introduction of ART into Barbadian healthcare [3].

It was notable that VL levels in untreated HIV were relatively low and that these improved further during the years 2009–2011. The low VL set-point may be due to the impact of an unusually high frequency (27% of the population) of protective HLA alleles (B27/57/5801) capable of reducing viral fitness in the Barbados population and this is the subject of active ongoing investigation [40]. Further suppression of VL during the years 2009–2011 may have been due to two reasons: either, improved quality of treatment of co-infection or cardiometabolic disease in patients who had entered the program, that may have slowed viral progression (since inflammation and immune activation drives viral replication) [41]; [42], and/or, raising of the CD4 treatment threshold in 2010 from 200 to 350 cells/mL, hence removing the sickest patients from the untreated client pool. Whatever the reason, these data suggest that our 26% projection of population level HIV suppression in 2010 may have been conservative, since that estimate was restricted to patients on ART and did not count this treatment naïve population with suppressed VL.

Finally, we note that ongoing VL surveillance is a powerful monitoring and evaluation (M&E) tool for the Ministry’s national HIV program. This HIV program is being transitioned from the current model, centered on a dedicated facility, into outlying primary health care clinics as part of a strategy to ensure the sustainability of the HIV response. Monitoring of VL suppression will provide an important and objective marker of personal as well as public health outcomes to evaluate the performance of the national HIV response as it undergoes this transition to primary health care.
